# Sentimental and spatial analysis of COVID-19 vaccines tweets

**DOI:** 10.1007/s10844-022-00699-4

**Published:** 2022-04-15

**Authors:** Areeba Umair, Elio Masciari

**Affiliations:** Department of Electrical Engineering and Information Technologies, University of Naples Federico II, Via Claudio, Naples, 80125 Campania Italy

**Keywords:** Sentimental analysis, COVID, Vaccines, Vaccine hesitancy, Spatial analysis

## Abstract

The world has to face health concerns due to huge spread of COVID. For this reason, the development of vaccine is the need of hour. The higher vaccine distribution, the higher the immunity against coronavirus. Therefore, there is a need to analyse the people’s sentiment for the vaccine campaign. Today, social media is the rich source of data where people share their opinions and experiences by their posts, comments or tweets. In this study, we have used the twitter data of vaccines of COVID and analysed them using methods of artificial intelligence and geo-spatial methods. We found the polarity of the tweets using the TextBlob() function and categorized them. Then, we designed the word clouds and classified the sentiments using the BERT model. We then performed the geo-coding and visualized the feature points over the world map. We found the correlation between the feature points geographically and then applied hotspot analysis and kernel density estimation to highlight the regions of positive, negative or neutral sentiments. We used precision, recall and F score to evaluate our model and compare our results with the state-of-the-art methods. The results showed that our model achieved 55% & 54% precision, 69% & 85% recall and 58% & 64% F score for positive class and negative class respectively. Thus, these sentimental and spatial analysis helps in world-wide pandemics by identify the people’s attitudes towards the vaccines.

## Introduction

In December 2019, Coronavirus started spreading a infectious diseases, called COVID-19 in Wuhan, China (Adamu et al., 2021; Huang et al., 2020). COVID-19 was declared as pandemic by WHO (World Health Organization) in March 2020 when it affected the whole world. Statistics on Worldometer (https://www.worldometers.info/coronavirus/, accessed on 17 November 2021), shows that 255,551,120 number of people have been affected by COVID and it has caused 5,135,962 number of deaths. Various preventive measures were adopted by different countries but the permanent solution of this problem was the invention of its vaccines for long-term protection (Chou & Budenz, 2020; Umair et al., 2021). Different BioTech industries, research universities and pharmaceutical staff tried their best for developing the COVID-vaccine. After development of vaccines, its acceptance among the people was another challenge (Kourlaba et al., 2021). The maximum delivery of vaccines all over the world can result in the control of covid (Seale et al., 2020; Freed, 2021). Due to fear of side-effects and other rumours, most of the populations is not willing to accept it (Green et al., 2021; Hogan et al., 2020; Lazarus et al., 2021). Therefore, the government and other agencies need to analyse the people’s sentiments of vaccine campaigns so that maximum vaccine receiving can be made sure (Seale et al., 2020; Sv et al., 2020).

Previously, data available was a big challenge for the researchers but with the development of the social media platforms, the user-generated data is freely available to use (Umair et al., 2020; Flesca et al., 2007). People use different social media platform to share their thoughts and feelings or to express their opinion about the current topics (Basile et al., 2021). This user-generated free data can be used positively to analyse the public opinion in many aspects. Social media big data can be very helpful in investigating the sentiments of people, their behavior throughout the duration of disease and the precautionary measures they were adopting to avoid the pandemic (Jelodar et al., 2020; Zhou et al., 2020). Out of all other social media platforms, twitter is a famous social network which allows its users to express their opinion about anything in the form of tweets or comments (Das & Dutta, 2020) and (Samuel et al., 2020b). These tweets can help agencies or policy makers to analyse the people’s feedback on any current topic (Luo & Xu, 2021). The famous methods for analysing the online contents are machine and deep learning (Jelodar et al., 2020). Sentimental analysis is a famous methods for this purpose (Manguri et al., 2020). The main part of sentimental analysis is to find the polarity (positive, negative or neutral) of the text or tweets (Raheja & Asthana, 2021).

Mapping is considered as critical tool in establishing the relationship between infectious disease control and their modern environment. Today, GIS based modeling is performed using digital as well as electronic big data (Zhou et al., 2020). Although the techniques have been evolving day by day, but the idea of spatial patterns of incidence with their environment remains same (Koch, 2016; Fazzinga et al., 2018). Vaccine data sharing is the foremost step in preparing, controlling monitoring and recovery of disease. As infectious disease phenomena are greatly related to spatial and temporal factors. Web based GIS have provided opportunities to visualize disease control and vaccine information over maps. The web-based tools have caused a revolution in the history of disease mapping and controlling using - systems (GIS). The big electronic and print data can be visualized in interactive and real time dashboards, which can help to protect human lives. During Covid-19 vaccine campaigns, the live dashboards can be the pivotal source of information world-wide (Boulos & Geraghty, 2020).

In this research, we worked on analyzing the people’s feelings and sentiments which they have expressed on twitter about the vaccination campaign of COVID. The purpose of analysis is to help the government and other health-concerning departments to consider the opinion of the public while designing their vaccination policy. In the proposed model, we obtained the twitter data-set from the Kaggel website and then transform it into useful form using pre-processing steps. Then, we used TextBlob() function to get the polarity values of text. We further designed the wordcloud using the polarity and performed the sentimental classification using BERT model. At the end of this study, we visualized the COVID-vaccine data geo-graphically and applied various geo-spatial approaches such as hotspot analysis, kernel density estimation etc. in order to analyse the vaccine data geo-graphically. The purpose of conducting the research was:

- Use TextBlob to categorize the text seven classes based on their polarity and design the word-clouds using polarity.
- To classify the tweets on the basis of their polarity using BERT model.
- To perform the spatial analysis of vaccines related data using Geo-spatial approaches.

The structure of this research article is as follows: Section 2 presents the state-of-the-art of sentimental analysis during COVID-19 field. The overall methodology of the proposed scheme is explained in Section 3 while Section 4 describes the discussion on the results. Discussion on geo-spatial analysis of COVID tweets have been provided in Section 5. Finally, Section 6 concludes our study and discuss the future work as well.

## Related work

To use the NLP in order to analyse the opinion and emotions of a person is called sentimental analysis (Shofiya & Abidi, 2021). In recent past, many researchers tried to analyse the social media data and found sentiments of people. There exists several techniques to analyse the sentiments of people using dictionary-based methodology and corpus-based methodology (Ajantha Devi & Nayyar, 2021), using clustering method (Flint et al., 2021), using correlation analysis (Huang et al., 2020).

Now-a-days, COVID-19 is the biggest issue around the world. Considering this, many researchers are trying to analyse the people’s emotions from different perspective using available data, tools and techniques (Singh et al., 2021). In Adamu et al. (2021), researchers used the methods of KNN, RF, NB, SVM, DT and LR for sentimental analysis of palliatives distribution during COVID days using twitter dataset containing 9803 Tweets. Agarwal et al. performed the Mental Health Analysis of students by NLP using 330,841 tweets (Agarwal et al., 2021) while (Das and Dutta, 2020) extracted 410,643 tweets and used Scatter plot, line chart, LDA for public sentiments analysis during the lockdown. In Flint et al. (2021), the researcher understand adults’ thoughts and behaviors using k-means clustering algorithm. For this purpose, they performed the online survey to gather the data. Haung et al. constructed a framework of COVID-19 from five Dimensions i.e. epidemic, medical, governmental, public, and media responses using data from Weibo account and WeChat account. They performed the correlation analysis for this purpose on the extracted dataset (Huang et al., 2020). Another research direction during covid was to identify dominant topics during COVID using sentimental analysis using Latent Dirichlet Allocation (LDA) (Hung et al., 2020). In another study, Jelodar et al. used LSTM to uncover issues related to COVID-19 from public opinions. They used the data from Reddit containing 563,079 Comments (Jelodar et al., 2020). In Luo and Xu (2021), the researchers used 112,412 reviews from Yelp and analyse online restaurant reviews. They used the methods of GBDT, RF, LSTM, SWEM for sentimental analysis. The examination of worldwide trends of fear, anger, sadness, and joy was performed by Lwin et al. (2020) using 20,325,929 tweets from twitter. TextBlob is used to determine polarity and subjectivity in COVID 500,000 number of tweets (Manguri et al., 2020).

In another study (Nguyen et al., 2020), researchers analyzed the racial sentiments during COVID-19 using SVM model. Praveen et al. (2021) used Indian tweets in order to discuss attitude of Indian citizens while discussing the anxiety, stress, and trauma using TextBlob and LDA. In Raheja and Asthana (2021), researcher extract the subjectivity and polarity of 370 tweets and also draw the WordCloud using sentiments from the tweets. TextBlob, CNN-LSTM, RF, SVC, ETC, DT were used in Rustam et al. (2021) to perform Covid-19 tweets sentiment analysis. Another direction of research revolves around the sentiments of COVID patients using TF–IDF and LDA. They extracted 55,612 PORs of 3430 doctors from RateMDs, HealthGrades, and Vitals for this purpose. Some researchers also worked on examination of public discourse and sentiment regarding older and COVID-19 and assessed the extent of ageism using LR, SVM, RF, LDA in Xiang et al. (2021). In Yin et al. (2020), researchers used Dynamic Topic Models (DTM) to detect topic and sentiment dynamics due to COVID-19 pandemic on 13 million tweets. Zhu et al. used Qingbo Big Data Agency dataset and performed LDA to analyse social media topics and emotional change characteristics from spatiotemporal perspectives (Zhu et al., 2020).

After lockdown, everyone was craved for the reopening. Some researchers developed the understanding of the factors driving post-COVID-19 reopening sentiment using binary logit model on 293,597 tweets in Mokhlesur Rahman et al. (2020). Samuel et al. (2020a) also worked on the reopening sentiments after lockdown using N-gram approach. Singh et al. used BERT model to analyse the impact of corona virus in social life using their sentiments (Singh et al., 2021). Hesitancy of vaccine is another big challenge faced by all over the world. Many researchers are trying to decode the hidden sentiments of people related to vaccine available on social media. In Müller and Salathé (2020), researcher proposed concept drift on vaccine sentiments using BERT model. In Hussain et al. (2021), Hussain et al. utilied the methods of artificial intelligence and performed analysis of people’s attitudes and behaviour towards the vaccine using Facebook posts and tweets. They limited the scope of their study towards United States and United Kingdom. However, there is a need to perform the sentimental analysis on the vaccine data using some advanced methods of NLP and also classify the sentiments in different categories.

## Methodology

In literature, various manual and machine learning based approaches are used for text classification. Manual approaches work on the basis of defined rules while the machine learning approaches use algorithms for classifying the sentiments. We have used hybrid approaches in our work.

### Proposed scheme

**Fig. 1:**
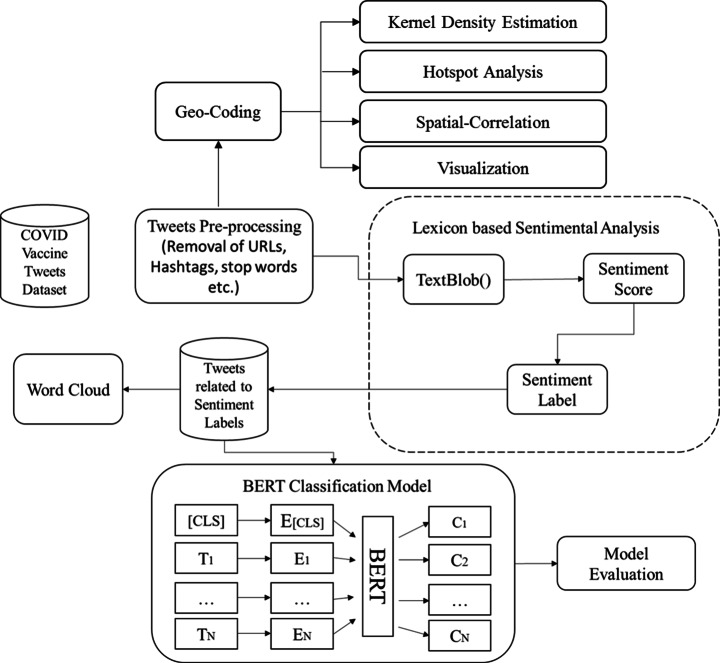
The scheme used in sentimental and spatial analysis of people’s reactions

Our methodology consist of five stages as shown in Fig. 1.

Stage one consists of dataset collection and pre-processing. Social media data is usually in unstructured form.

Stage two includes finding the semantic polarity of the extracted sentiments.

Stage three shows that most frequent buzz words found in the data are in the form of wordcloud.

Stage four is sentimental classification using BERT model.

Stage five is the geo-coding of the data and the spatial analysis of the tweets considering the sentimental polarity of the tweets.

### Dataset collection and pre-processing

**Fig. 2:**
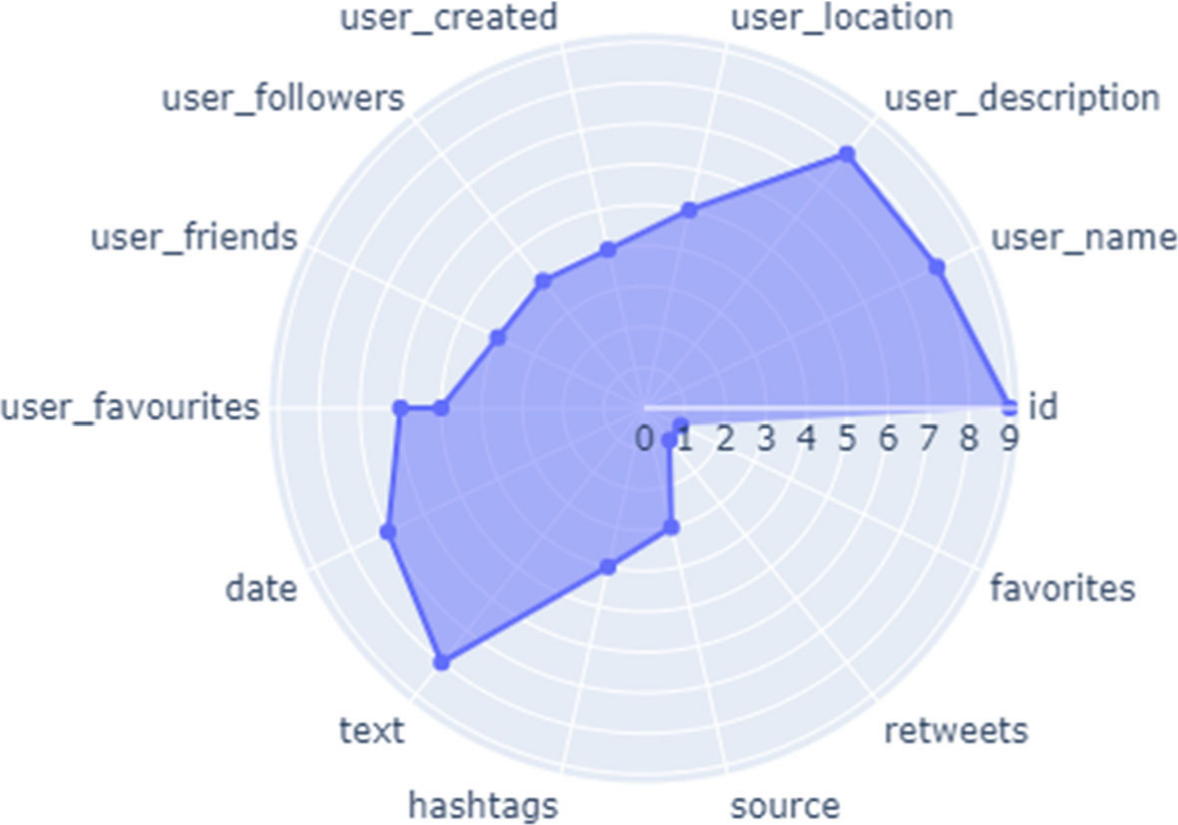
Exploratory data analysis

We used COVID-vaccine dataset of tweets which is freely available on Kaggle website in our work. The dataset contains the tweets from the entire world upto early May 2021. The Exploratory Data Analysis is given in Fig. 2

Then, the pre-processing of dataset was performed and we removed the hashtgs, URLs and stop-words form the tweet text. We used different modules of Python in order to perform the pre-processing. Regular expression module is very powerful while preparing the data for further analysis. In Table 1, tweets after applying pr-processing have been shown.

**Table 1:** Text before pre-processing and after pre-processing

Samples before pre-processing	Samples After removal of hashtags	Samples after removal of URLs
Got my second dose #COVID https://t.co/yt3vn67mVg	Got my second dose COVID https://t.co/yt3vn67mVg	Got my second dose COVID
We can see the better days in future #Pfizer https://t.co/77u4f8XXfx	We can see the better days in future Pfizer https://t.co/77u4f8XXfx	We can see the better days in future Pfizer
Caught COVID even after vaccine #Vaccine https://t.co/uQ3A2f7SVP	Caught COVID even after vaccine Vaccine https://t.co/uQ3A2f7SVP	Caught COVID even after vaccine Vaccine

### Sentiment polarity using TextBlob

It is important to know how much the given text is negative or positive for sentimental classification. Based on the polarity values, we have categorized the tweets into seven classes. Out of these seven classes, three are sub-classes of positive such as weakly, mild and strongly (positive), three are sub-classes of negative such as weakly, mild and strongly (negative), and the last one is neutral (Singh et al., 2021). We defined the sentiment range for each class using the rules of Singh et al. (2021).

According to the rules defined in Singh et al. (2021), the text is considered as neutral if its polarity is found to be 0. If the polarity value of the text is greater than 0 and <= 0.3, the text is classified as weakly positive. Similarly, if the polarity range is greater than 0.3 and <= 0.6, the identified class will be mild positive. The text is classified as strongly positive if falls between 0.7 and 1. Similarly, the weakly negative class will be identified if the polarity values is greater than − 0.3 and <= 0 The mild negative class is given if polarity > − 0.6 and <= − 0.3. However, the strongly negative text should have the polarity values > 1 and <= − 0.6.

**Fig. 3:**
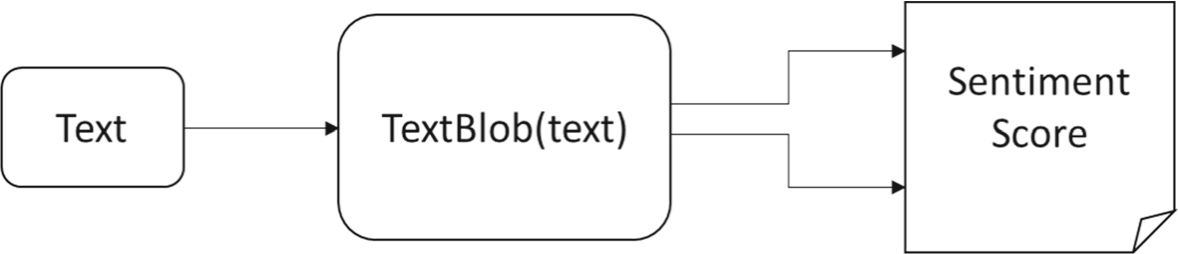
TextBlob() working

We found out the polarity of the tweets using library functions of Python. One of these functions is TextBlob() which gives the polarity between [− 1 to + 1]. The working of TextBlob() has been shown in Fig. 3.

### Social network analysis

The text of tweets contains words with different intensities. Some words are repeating again and again and hence their frequency is higher. We can identify the high frequent words in each class i.e. neutral, negative and positive class. These words can form a wordcloud in which size of each word represents their frequencies. If a word is biggest, it means that its frequency in the given text is highest among all other words. We plotted three word clouds to see the most prominent and expressive words related to COVID vaccine tweets. Such type of word cloud are the crucial methods to identify the public’s feelings and expressions in a quick manner.

We converted the dataset into three subsets of positive, negative and neutral datasets based on the polarity labels. We used numPy and Pandas library of Python to read these files separately. For visualization, matplotlib Python library is used which helps many other libraries including wordcloud to plot the results. The only required thing to form the wordcloud is text. The three steps are taken for the wordcloud formation.

- Load the input data i.e. text
- Generate the wordcloud image
- Display the wordcloud image in the screen

We also applied the masking in order to enhance the visualization of the clouds. The results of the wordcloud are given in Section 4.2.

### Sentimental analysis of people’s tweets

BERT is a language model which is a transformer-based ML model, helps to carry out natural language tasks. It is a deep learning model in which every output element has a direct connection with almost every input element. Keeping in view the application of BERT model, we have it in our classification task for COVID-19 vaccine. Its architecture is most accepted among all of the language models (Pota et al., 2021; Yadav and Singh, 2020). We used BERT base architecture in this research.

#### BERT architecture

**Fig. 4:**
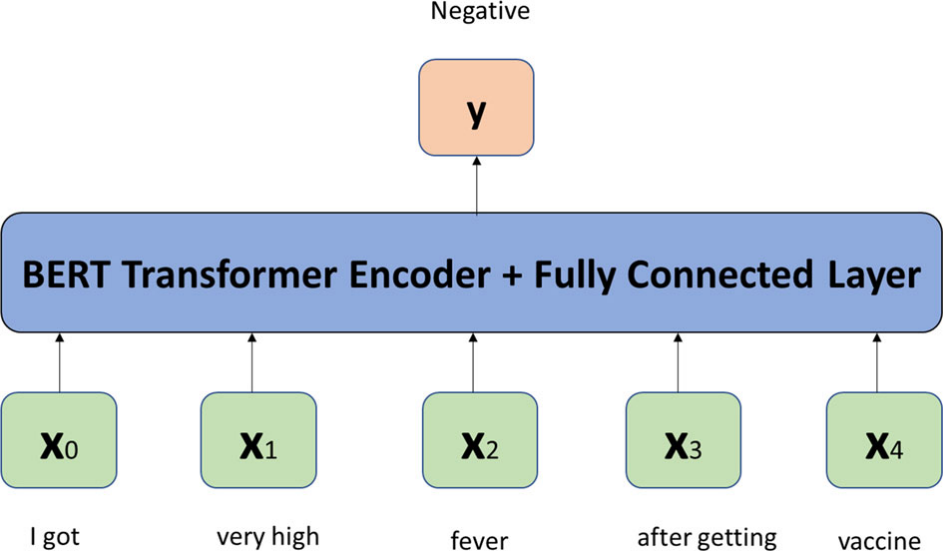
BERT system

BERT is based on the transformer architecture and uses unlabeled data for pre-training. Figure 4 shows the architecture of BERT, where the outermost layer is used to refine the text keeping the internal layer parameter idle. The text body is helping to train the core architecture.

The special tokens, that are found in BERT for segment separation and classification are [SEP] and [CLS] respectively. The first input of classifier is represented by these tokens. The probability of the classes can be found using the (1).

1 $$  P = softmax(CW^{T})  $$

##### Transformer

A typical transformer is the encoder-decoder network, where the self-attention is used on the encoder end while attention is used on the decoder end. The transformer architecture consists of self-attention layer and layers for encoder and decoder. The layers of encoder consists of two sub-layers that are feed forward and multi-head self attention mechanism (Blaauw & Bonada, 2019; Devlin et al., 2019). While decoders layer has three sub-layers i.e. feed forward, multi-head self attention, multi-head self-attention layer on output of an encoder (Dong et al., 2018; Furfari(tony), 2002). The layer normalization and residual connection have also been applied on both encoder and decoder (Chen et al., 2020). Attention is the mapping of keys and values to outputs (Moritz et al., 2020; Liu & Lapata, 2020; Shin et al., 2019). The attention can be computed using formula given in (2).

2 $$  Attention(Q, K, V)=SoftMax \left( \frac{QKT}{\sqrt{dv}}\right) V  $$

#### Experiments

In experiments, we used Adam optimizer as a loss function. Adam optimizer helps in model training by updating the parameters and keep output to the optimal values (Liu, 2021). The hyper-parameter along with their values are given in the Table 2.

**Table 2:** Parameters along with their values

Parameters	Value
BATCH_SIZE	16
LEARNING_RATE_MODEL	1e-5
LEARNING_RATE_CLASSIFIER	1e-3
EPOCHS	5
WARMUP_STEPS	0
GRADIENT_ACCUMULATION_STEPS	1
MAX_GRAD_NORM	1.0
SEED	42
NO_CUDA	False

We tuned two different models for positive and negative sentiment classification. The matrices of Recall (4), Precision (3) and F_1_ score (5) were used for model evaluation.

3 $$  Precision = \frac{True Positive}{True Positive + False Positive}  $$

4 $$  Recall = \frac{True Positive}{True Positive + False Negative}  $$

5 $$  F Measure = \frac{2 \times Precision \times Recall}{Precision + Recall}  $$

#### Comparison of our approach with state-of-the-art

To evaluate the performance of the proposed scheme, few state-of-the-art machine learning algorithms have been chosen based on their usage in literature including K-nearest neighbour (KNN), Naive Bayes (NB), Random Forest (RF), Support Vector Machine (SVM) and Decision Tree (DT) (Adamu et al., 2021; Jelodar et al., 2020; Rustam et al., 2021).

Random Forest only considers user interest and preferences (Yi et al., 2018). Random Forest works on the basis of votes collected from classification and regression trees. It chooses random samples from the attributes as well as instances of the dataset (Rangnekar et al., 2018). Decision tree is usually used on the simple dataset and does not consider good for complex data because it works on simple decision rules found in the entire dataset (Almanie et al., 2015).

Naïve Bayes gives equal importance to each factor and works on the probability based on Bayesian theorem given in (6): (Almanie et al., 2015; Abdulrahman & Abedalkhader, 2017).

6 $$  P (H|X) = P (X|H) P (H)/ P (X)  $$

KNN is the algorithm which does not train the model but it just finds most similar values based on the neighbours (Abdulrahman & Abedalkhader, 2017). KNN searches for the most similar sample by computing the distance given in the formula (7).

7 $$  di = \sqrt{[(xi-x)^{2}+(yi-y)^{2}]}  $$

SVM finds the hyper-plane and converts the feature space into high dimensional features. It uses verious kernel functions such as linear, polynomial, sigmoid, and radial basis function (RBF) to work (Liu et al., 2019).

## Results and discussion

This study has highlighted the sentiments of people towards COVID-19 vaccine in all the regions of the world. The viral infections are not territoriality bounded, therefore there is sheer need to perform the sentimental and spatial analysis, which uses artificial intelligence methods to monitor the areas of positive, negative or neutral sentiments. This study performed sentimental analysis using BERT, drew word cloud and performed the GIS based analysis to examine the present and future consequences of the COVID-19 vaccine hesitancy throughout the whole world.

### Sentiment polarity

The polarity helps to identify the sentiment of the text. In Table 3, we have presented the sample tweets along with their polarity and also mentioned the categories of each tweets with respect to their sentiment as defined in Section 3.3

**Table 3:** Sample tweets along with their polarity and sentiment categories

Tweet sample	Polarity	Sentiment
Got my second dose COVID	0	Neutral
We can see the better days in future Pfizer	0.675	Strongly positive
Caught COVID even after vaccine	− 0.25	Weakly negative

### Word cloud

**Fig. 5:**
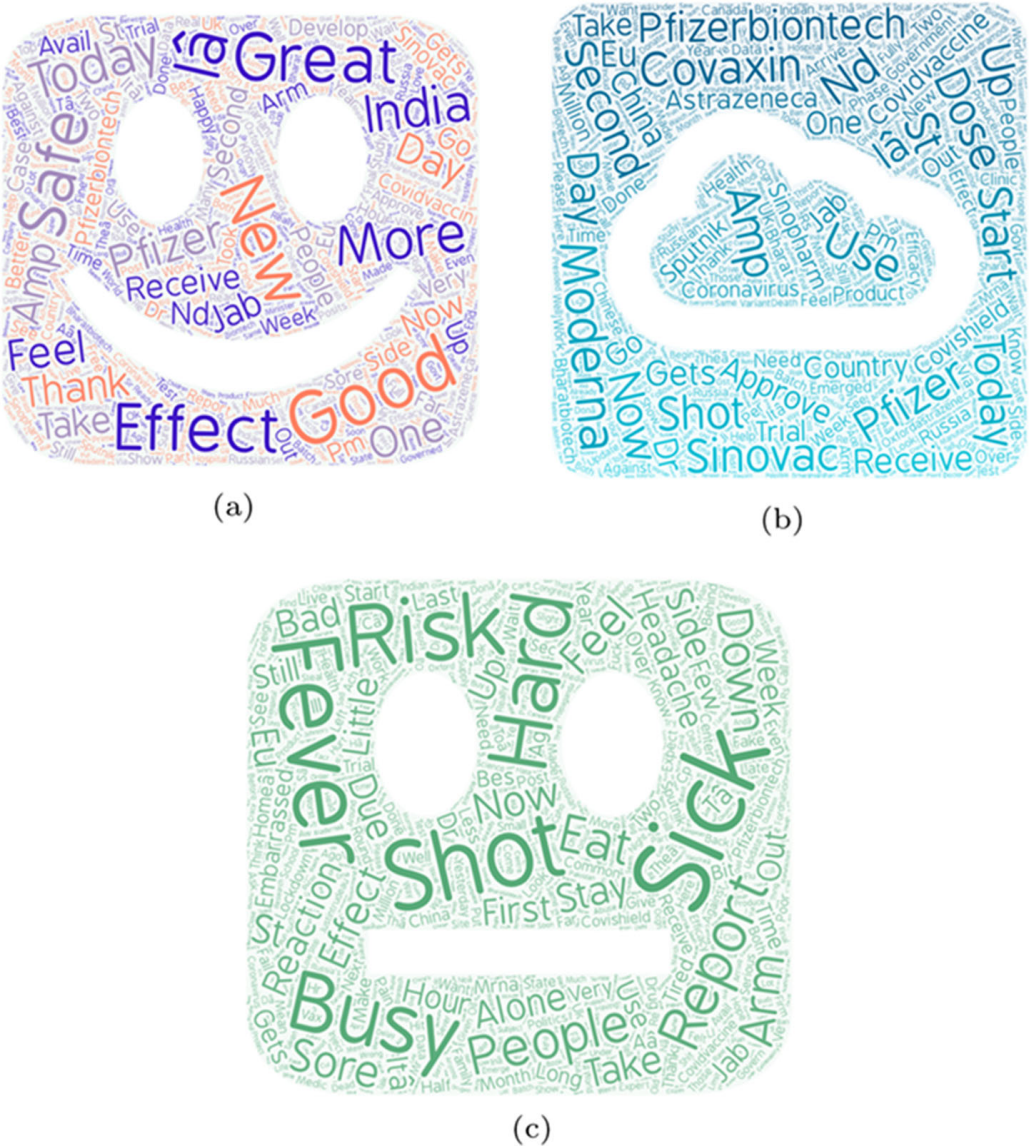
Figure (a) shows the word cloud of positive words, (b) shows the word cloud of neutral words while (c) shows the word cloud of the negative words

We plotted the word cloud using the tweet text, considering three categories i.e. neutral, positive and negative. Using the polarity values, we first created three different data sets for each category respectively. Figure 5 illustrated the three word clouds such as neutral cloud, negative cloud and positive cloud. The groups of words which fall into particular category are given below:

- Words like “Great”, “Good”, “More”, “Safe”, “Thank”, “Better”, “Happy”, “Love” represent the positive attitude of the people towards the vaccine.
- Words Like “Headache”, “Fake”, “Fail”,“Sick”, “Fever”, “Risk”, “Down”, “Sore” are categorized as negative and they represent the negative feedback of people towards vaccine.
- Words such as “Today”, “Start”, “Use”, “Now”, “China”, “Effect”, “shot”, “second”, are neither positive nor negative.

### Sentimental classification

**Fig. 6:**
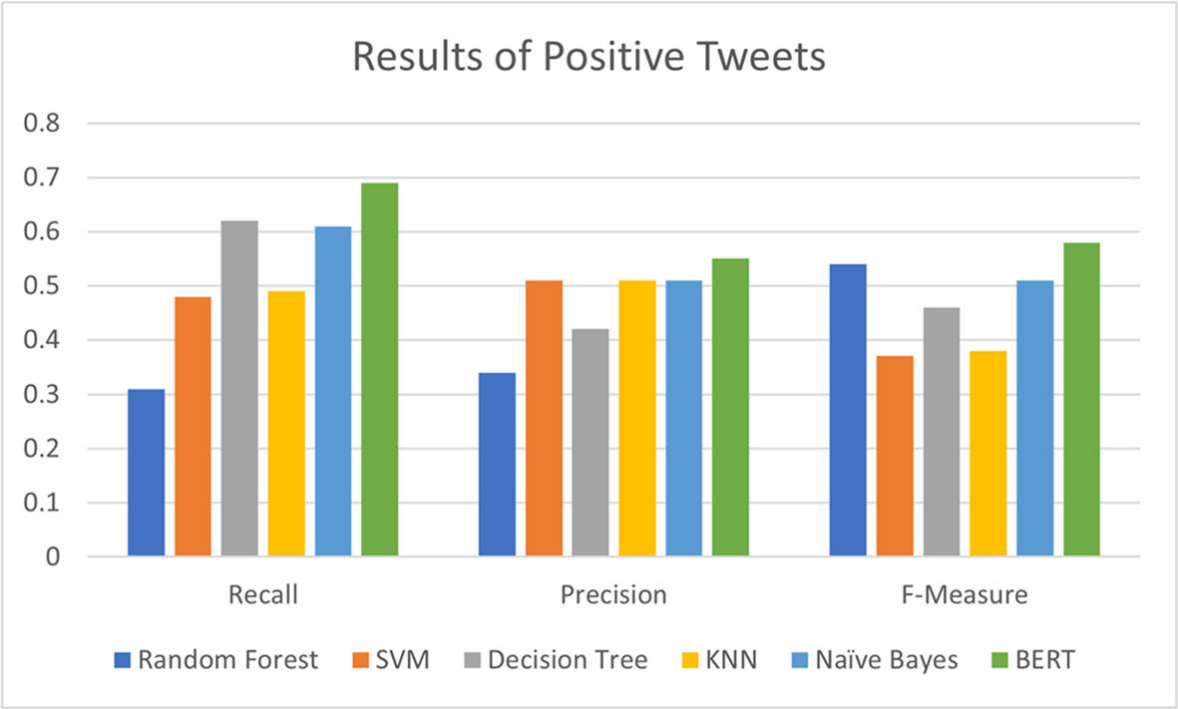
Performance of BERT model as compared to state-of-the-art for positive sentiment classification

**Fig. 7:**
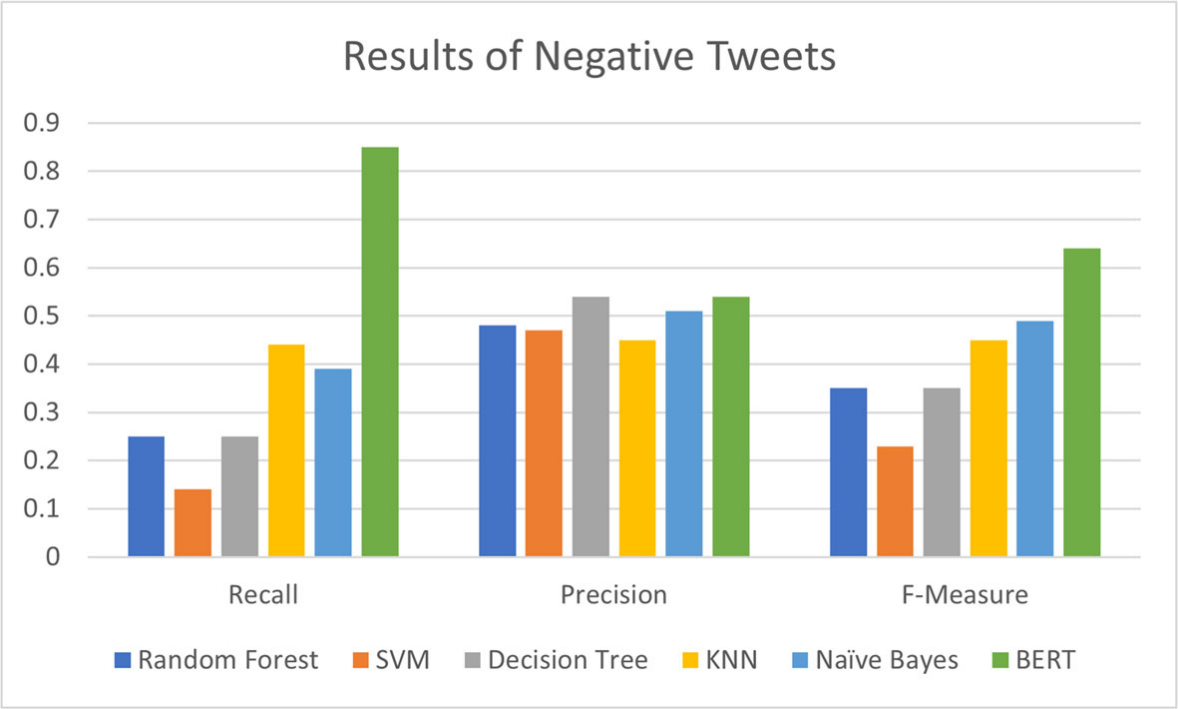
Performance of BERT model as compared to state-of-the-art for negative sentiment classification

The results of comparison of BERT model with the state-of-the-art have been shown in Figs. 6 and 7.

We can see from the Figs. 6 and 7 that our proposed BERT model outperformed the state-of-the-art machine learning model for positive as well as negative sentiment classification by achieving maximum precision, recall and F-measure. BERT achieved 55% precision, 69% recall and 58% F-score in the case of positive tweet classification while it achieved 54% precision, 85% recall and 64% F-score for negative tweet classification. These results are highest amongst all other state-of-the-art algorithms.

### Strengths and limitations of our analysis

The data from only one social media platform can not be enough for analysis on such worldwide issues because different social media applications have their own popularity in different parts of the world (Singh et al., 2021; Umair & Masciari, 2021). The negative messaging spread the hate about vaccine, such type of messages need to be identified (Chou & Budenz, 2020). Hence, data from Facebook, WeChat, Instagram should be merged with that of twitter in order to analyse the worldwide sentiments of the people (Pota et al., 2021). Moreover, different countries of the world use their own national languages other than English. They communicate, write, post, review in their own languages i.e. China, Italy etc. Therefore, there is a need to develop model which translate the posts in other languages as well so that worldwide sentimental analysis can be performed.

## Discussion on geo-spatial analysis of COVID vaccine tweets

Today, GIS based modeling is performed using digital as well as electronic big data (Zhou et al., 2020). Although the techniques have been evolving day by day, but the idea of spatial patterns of incidence with their environment remains same (Koch, 2016). Disease data sharing is the foremost step in preparing, controlling monitoring and recovery of disease. As infectious disease phenomena are greatly related to spatial and temporal factors. Web based GIS have provided opportunities to visualize disease information over maps. The web-based tools have caused a revolution in the history of disease mapping and tracking using geoinformation systems (GIS). The big electronic and print data can be visualized in interactive and real time dashboards, which can help to protect human lives.

### Geo-coding and visualization of data

**Fig. 8:**
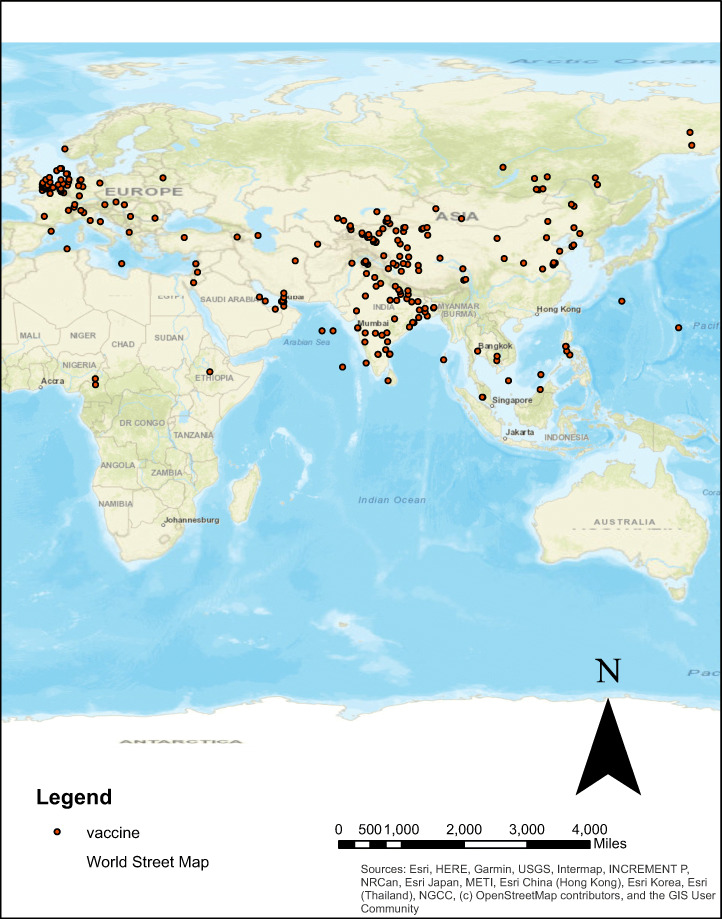
Visualization of vaccine data on world map

We have created a subset of data and performed Geo-coding using Geo-Py module of Python. Geo-py is used to locate the coordinates of landmarks, locations, address, cities and countries around the globe using third-party geo-coders (Chong & Chen, 2021). Vaccine tweets data was visualized using ArcGIS 10.5. The mapping of data over topological map displays the distribution of COVID-19 vaccine worldwide. Figure 8 shows the vaccine distribution in the world.

### Analyzing the patterns

Analyzing the patterns gives the overview of the relationship between the features of the dataset. The features can either be clustered, random or dispersed. There exist the patterns in data if the data falsified the null hypothesis (i.e. the features are in complete state of randomness). On the other hand, it exhibits the relationship of either clustered or dispersed. The clustered relationship is important because it shows the high geographical associativity between the features.

#### Average nearest neighbor

Average nearest neighbor is the tool that helps to identify the spatial relationship of the features present in the dataset. Average nearest neighbor returns a ratio of observed average distance to the expected average distance as shown in (8).

8 $$  ANN = DO / DE  $$

We have applied the ANN tool over vaccine tweet dataset to find if either the feature set is geographically significant of not.

**Fig. 9:**
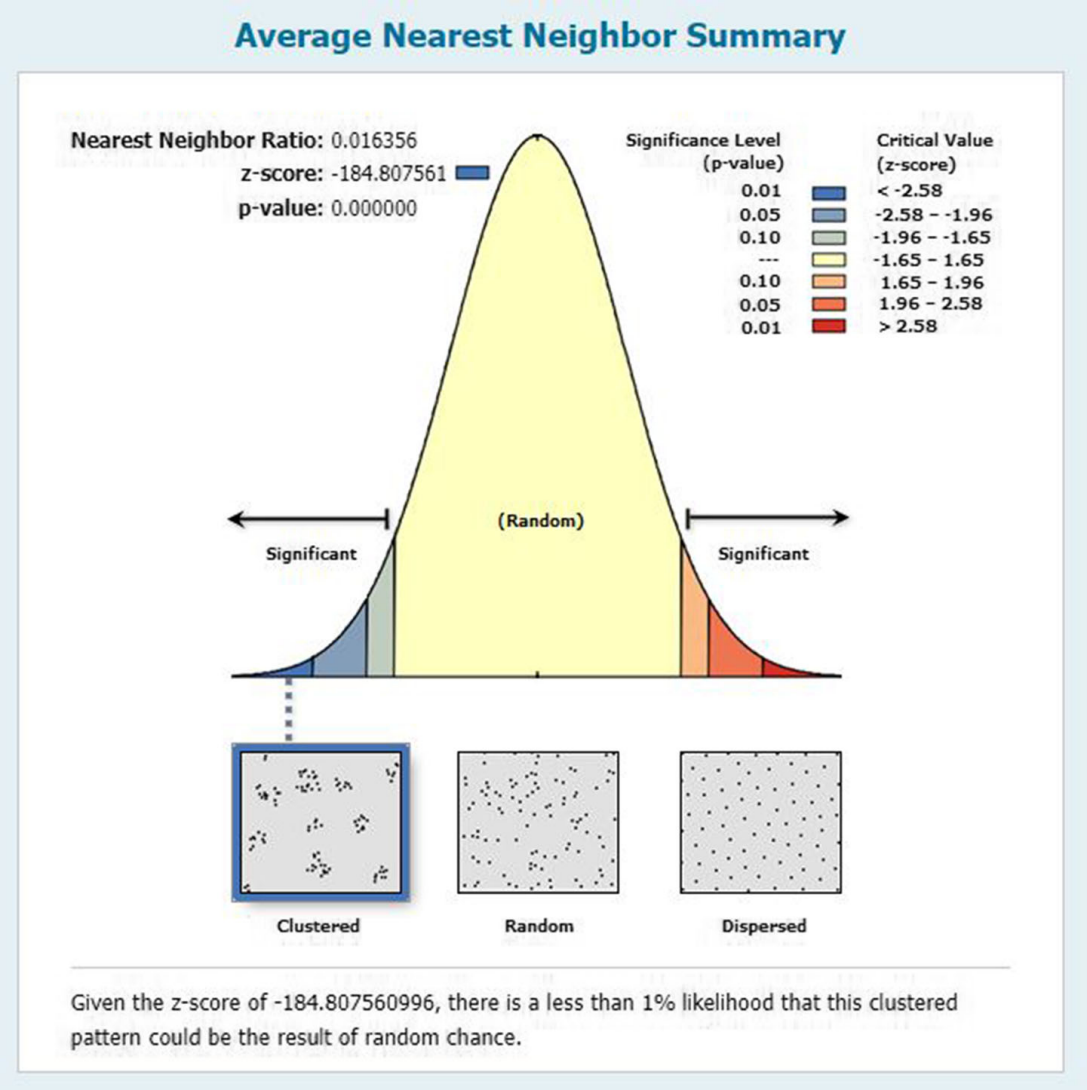
Spatial relationship of vaccine data using ANN

Figure 9 shows that our tweets data is scientifically clustered which means that we can perform certain spatial operations on the data.

### Hotspot analysis

Hotspot shows the geographical areas (Tabangin et al., 2008) where the vaccine sentiment polarity is high in rate while cold spots shows the areas with less vaccine sentiment polarity. Creating hotspots in maps help to better investigate the sentiments of the people toward vaccine. We used the Hotspot analysis tool of ArcGIS software for this purpose. It works based on the polarity values of each tweet.

**Fig. 10:**
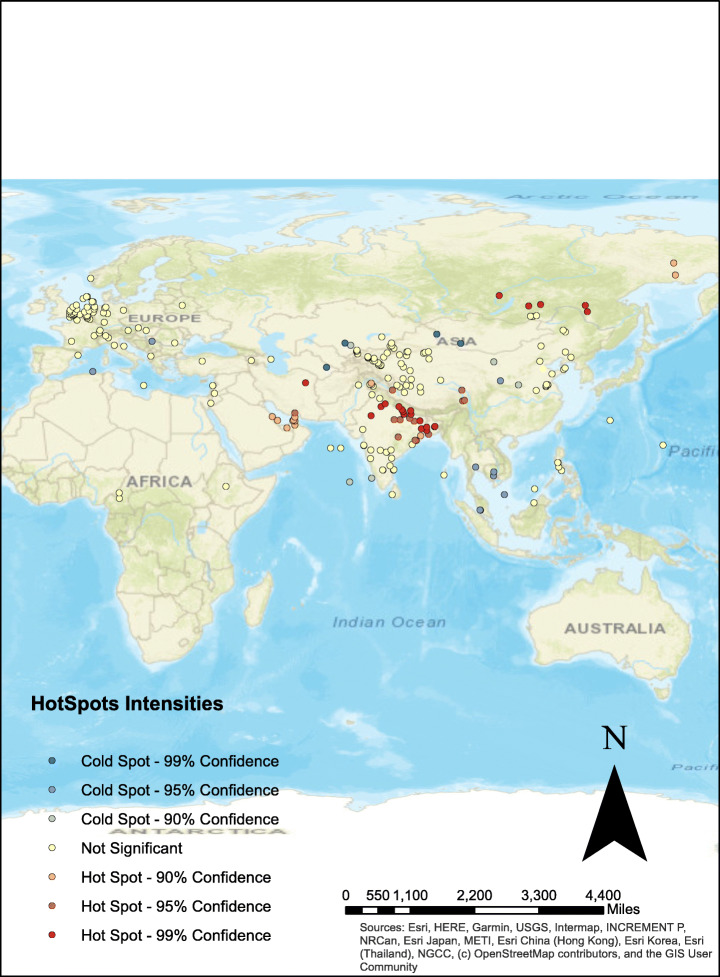
Hotspot analysis of COVID-vaccine tweets

It is clearly visible from the Fig. 10 that Asia countries such as India, Saudi Arabia are showing more positive attitude towards the vaccines while Europe is behaving neutral during vaccine. China and few other countries are showing negative sentiments for vaccine. According to a systematic review of vaccine acceptance rates in Sallam (2021), higher-income, age and gender are the main reason behind the different behaviors of people in different regions of the world.

### Analysis using kernel density estimation

**Fig. 11:**
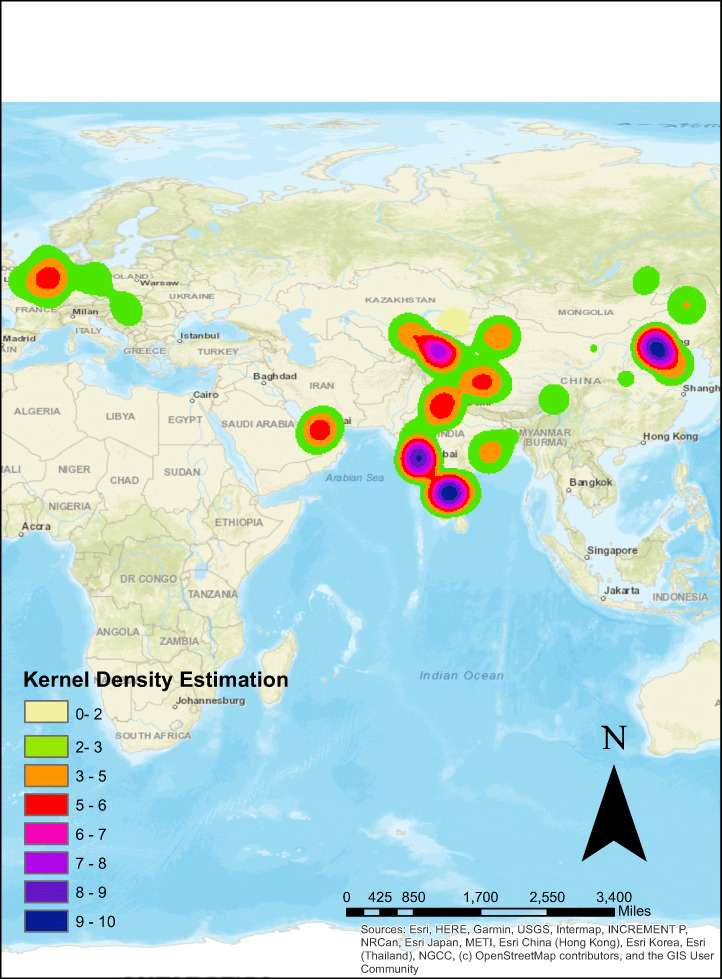
Kernel density estimation of COVID-vaccine tweets

Point density can be envisioned as the series of circles around each feature point, and the density being calculated as the number of circles being over-lapped (Haan, 1999). Kernel density can be envisioned as putting the blob of ice-cream on the top of each feature point and then the density function be the measuring the height of the accumulated blobs. It interprets COVID vaccine data and extract valuable information for COVID modelling. We used the kernel density tool of ArcGIS to perform density analysis. Figure 11 represents the clusters formed using kernel density over COVID Vaccine dataset.

Figure 11 gives more closer overview of the people’s sentiment all over the globe. It also shows that the more positive sentiment polarity is found in India and Europe behave neutrally in this context.

## Conclusion and future work

Twitter-based vaccines sentiment analysis is the valuable and easy implemented method in order to identify the vaccine sentiments among the people. Twitter analysis linking with the Geo-coded information helps to identify the reasons behind the different attitudes and behaviours of people at different regions. COVID-19 has emerged as a pandemic causing many people to be infected and has caused thousands of deaths worldwide. Besides preventive measures, the development of vaccines was the need of the hour. But the world is facing an even bigger challenge in the form of vaccine hesitancy. Our work focuses on identifying the sentiments of people about vaccines using the BERT model. For this purpose, we have used twitter data about vaccines and performed pre-processing steps. We found the polarity of tweets and categorized the tweets based on their polarity. We designed three word-clouds such as positive, negative and neutral clouds. Then, we fine tuned the BERT model for vaccine sentiment classification. Our work emphasizes over discovering the relationship of vaccine features geographically. Modern GIS technologies enable us to visualize the current state of vaccines and visualize vaccine hesitancy on a large scale. We have identified the highly positive, negative and neutral regions using hot-spot analysis and kernel density estimation. Hence, such types of advanced methods are the effective way to analyse the hidden sentiments of the people in tweets, posts or reviews. In future, we will design live dashboard which real-time sentiments of people could be analyzed along with all the statistics.

## Data Availability

The datasets generated during and/or analysed during the current study are available from the corresponding author on reasonable request.
